# Attributing Effects of Aqueous C_60_ Nano-Aggregates to Tetrahydrofuran Decomposition Products in Larval Zebrafish by Assessment of Gene Expression

**DOI:** 10.1289/ehp.9757

**Published:** 2007-02-21

**Authors:** Theodore B. Henry, Fu-Min Menn, James T. Fleming, John Wilgus, Robert N. Compton, Gary S. Sayler

**Affiliations:** 1 The Center for Environmental Biotechnology; 2 Department of Chemistry; 3 Department of Microbiology and; 4 Department of Ecology and Evolutionary Biology, University of Tennessee, Knoxville, Tennessee, USA

**Keywords:** microarray, nano-C_60_, nanotoxicology, THF, toxicogenomics, zebrafish

## Abstract

**Background:**

C_60_ is a highly insoluble nanoparticle that can form colloidal suspended aggregates in water, which may lead to environmental exposure in aquatic organisms. Previous research has indicated toxicity from C_60_ aggregate; however, effects could be because of tetrahydrofuran (THF) vehicle used to prepare aggregates.

**Objective:**

Our goal was to investigate changes in survival and gene expression in larval zebrafish *Danio rerio* after exposure to aggregates of C_60_ prepared by two methods: *a*) stirring and sonication of C_60_ in water (C_60_–water); and *b*) suspension of C_60_ in THF followed by rotovaping, resuspension in water, and sparging with nitrogen gas (THF–C_60_).

**Results:**

Survival of larval zebrafish was reduced in THF–C_60_ and THF–water but not in C_60_–water. The greatest differences in gene expression were observed in fish exposed to THF–C_60_ and most (182) of these genes were similarly expressed in fish exposed to THF–water. Significant up-regulation (3- to 7-fold) of genes involved in controlling oxidative damage was observed after exposure to THF–C_60_ and THF–water. Analyses of THF–C_60_ and THF–water by gas chromatography–mass spectrometry did not detect THF but found THF oxidation products γ-butyrolactone and tetrahydro-2-furanol. Toxicity of γ-butyrolactone (72-hr lethal concentration predicted to kill 50% was 47 ppm) indicated effects in THF treatments can result from γ-butyrolactone toxicity.

**Conclusion:**

This research is the first to link toxic effects directly to a THF degradation product (γ-butyrolactone) rather than to C_60_ and may explain toxicity attributed to C_60_ in other investigations. The present work was first presented at the meeting “Overcoming Obstacles to Effective Research Design in Nanotoxicology” held 24–26 April 2006 in Cambridge, Massachusetts, USA.

Nanoscience focuses on investigations of phenomena at the nanoscale and is the foundation for nanotechnology, which develops practical applications for nanomaterials (U.S. Nanotechnology Initiative 2006). Because nanomaterials have unique properties and are potentially biologically active, there is significant concern that harm to ecosystem and human health could occur after environmental contamination. According to a recent report, carbon nanomaterials (fullerenes and nanotubes) have the highest relative frequency of occurrence in consumer products already on the market (Nanotechnology Consumer Products Inventory 2006), and these materials may contaminate the environment in the future (Colvin 2003).

Fullerenes (i.e., Buckminsterfullerene, or “Bucky balls”) are nanomaterials that gained attention after the first preparation of C_60_, a novel allotrope of carbon consisting of 60 carbon atoms joined to form a cagelike structure (Kroto et al. 1985). The unique structure of C_60_ facilitates absorption of light and transfer of this energy to triplet oxygen, thereby forming the highly reactive singlet oxygen state (Arbogast et al. 1991). High yield of singlet oxygen with consequential generation of free radicals suggests that presence of C_60_ in the environment may cause oxidative damage in exposed organisms.

Release of C_60_ into the environment may lead to contamination of aquatic ecosystems and presence of bioavailable nanoparticles. C_60_ is exceedingly insoluble in water (2 × 10^−24^ mol/L; Nakamura et al. 1996); however, nanoparticles (< 220 nm) consisting of aggregates of C_60_ can occur (Andrievsky et al. 1999; Scharff et al. 2004), and these aggregates are relevant for exposure in aquatic organisms (Lovern and Klaper 2006; Oberdörster 2004). Aqueous aggregates of C_60_ can be generated by mixing pure C_60_ in water or by use of vehicle solvents. Tetrahydrofuran (THF) has been used as a vehicle to generate aqueous aggregates of C_60_ (Deguchi et al. 2001) in toxicology studies (Lovern and Klaper 2006; Oberdörster 2004), and yields water with a persistent amber color (Fortner et al. 2005). However, THF can alter surface charges of C_60_ particles, and THF may be retained between adjacent C_60_ molecules within aggregates (Brant et al. 2005). Thus, there is potential that toxicity attributed to C_60_ (e.g., Lovern and Klaper 2006; Oberdörster 2004) could actually result from the presence of THF or THF degradation products (Brant et al. 2005). Following our initial presentation of the present research (“Overcoming Obstacles to Effective Research Design in Nanotoxicology,” Cambridge, Massachusetts, USA, 24–26 April 2006), subsequent studies (e.g., Oberdörster et al. 2006; Zhu et al. 2006) have prepared aqueous C_60_ aggregates without THF or other organic solvents.

The objective of the present research was to investigate toxicity of aqueous C_60_ nanoparticles in larval zebrafish *Danio rerio*, and to determine if THF or THF degradation products used to prepare aqueous C_60_ can be responsible for toxic effects. End points of toxicity included survival, behavior, and changes in global gene expression.

## Materials and Methods

### Fish

Zebrafish *D. rerio* were obtained from the Zebrafish Research Facility at the University of Tennessee in Knoxville, Tennessee, and all experiments were conducted with approval from the University of Tennessee Institutional Animal Care and Use Committee. In all experiments, zebrafish were treated humanely and with regard for alleviation of suffering. Water for holding fish and conducting experiments (designated “fish water”) was prepared with purified MilliQ water (Millipore Corp., Bedford, MA) with ions added: 19 mg/L NaHCO_3_, 1 mg/L sea salt (Instant Ocean Synthetic Seasalt, Mentor, OH), 10 mg/L CaSO_4_, 10 mg/L MgSO_4_, 2 mg/L KCl. The “fish water” had the following characteristics: pH 7.3–7.9; dissolved oxygen > 6 mg/L; total alkalinity, 30–40 mg/L (as CaCO_3_); and total hardness, 15–20 mg/L (as CaCO_3_). All larvae used in experiments were obtained (Westerfield 1993) at the same time and were the same age (i.e., fertilization occurred within 15 min for each embryo). Temperature was 27 ± 1°C and photoperiod was 14 hr of light.

### Chemicals

Pure (99.5%) C_60_ was obtained from SES Research (Houston, TX), and tetrahydrofuran (THF; HPLC grade) was obtained from Fisher Scientific (Pittsburgh, PA, USA). γ-Butyrolactone (purity > 99%) was obtained from Sigma Chemical Co. (St. Louis, MO).

### Preparation of exposure solutions

**Figure 1:**
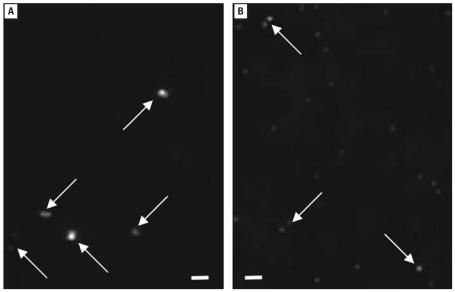
Photomicrographs of nanoparticles (arrows) in aqueous solution obtained by dark-field microscopy (100× objective, oil immersion) under identical conditions with enhanced resolution system. (*A*) Aqueous aggregates of C_60_ generated by THF vehicle (THF–C_60_). (*B*) FluoSpheres (Invitrogen, Carlsbad, CA, USA) (carboxylate modified microspheres, 100-nm diameter) in 2 mM Na_2_NO_3_ solution. Comparison of C_60_ aggregates indicates they were ≈ 0.5–3 times the size of the FluoSpheres. Scale bars = 1 μm.

Aqueous C_60_ aggregates were prepared by two methods: *a*) long-term (7 days) stirring and sonication of pure C_60_ in fish water (C_60_–water, 12.5 mg C_60_/500 mL); and *b*) use of THF as a vehicle. The procedure for generating aqueous C_60_ with THF was modified from a previously reported method (Deguchi et al. 2001; Fortner et al. 2005) as follows: approximately 12.5 mg of C_60_ was added to 500 mL of fresh THF in a 1-L amber bottle and sparged with UHP (ultra high purity) nitrogen to remove oxygen. The resealed amber bottles [THF–C_60_ and THF–water (vehicle control)] were stirred for 24 hr at ambient temperature. Fish water (350 mL) was added to each bottle and sparged with UHP nitrogen for 1 hr, then stirred for 24 hr at room temperature. A Büchi Rotavap system (Büchi Labortechnik AG, Flawil, Switzerland) was used to remove THF, in the dark, at 65°C, and the resulting solutions (THF–C_60_ and THF–water) were sparged with nitrogen for 2.5 days before use in toxicity tests. Solutions of C_60_–water were allowed to settle and were carefully pipetted to avoid resuspension of any visible aggregates before addition of fish water to prepare dilutions for exposures. The THF–C_60_ stock solution did not have any visible (naked eye) aggregates, and dilutions with highest concentrations of THF C_60_ had a visible amber color. Examination of THF–C_60_ solution with an enhanced dark-field microscopy system (Cytoviva, Auburn, AL) designed to resolve particles < 100 nm, demonstrated particle aggregates approximately equal to 0.5–3 times the size of latex nanoparticles of 100 nm examined under identical conditions (Figure 1A, B). This method of particle analysis evaluates particles as they appear in the exposure solution and avoids artifacts introduced by use of transmission electron microscopy methods, which can alter particle size upon drying (Thundat et al. 1993).

### Water chemistry analysis

All THF-treated water samples were analyzed for presence of THF with a Hewlett Packard 6890 gas chromatograph with a 5973N mass spectrophotometer (Agilent Technologies, Foster City, CA, USA) equipped with an inert ion source. A 1-μL volume of sample water was injected into a J&W DB-5MS column (30^m^ × 0.25 mm ID, 0.25-μm film thickness; Agilent Technologies). Helium (UHP grade) was used as a carrier gas, and a constant flow rate (0.6 mL/min) was maintained by electronic pressure control. Injection temperature was 280°C and splitless injection was employed. The oven temperature program started at 30°C, for 4 min, increased to 100°C at 5°C/min, for 2 min, then to 300°C at 50°C/min and maintained for 3 min. MS detection was monitored at full-scan EI mode (*m/z* 25–100). The intensity of ion *m*/*z* 86 was used for quantification of γ-butyrolactone.

### Fish exposures

Two dose–response toxicity tests were conducted simultaneously with the test to evaluate changes in gene expression. All fish were exposed in 400-mL glass beakers containing 100 mL exposure water, and exposure began when larvae were 75 hr of age and ended when larvae were 147 hr of age postfertilization. In dose–response toxicity tests, each beaker contained 9–13 larvae and the following concentrations of either THF–C_60_ or THF–water: 0%, 1%, 5%, 10%, 20%, and 25% (vol/vol). In the test conducted to evaluate changes in gene expression, each beaker contained 37–45 larvae and the following treatments (each with six replicate beakers) were tested: control; C_60_–water (100%); THF–C_60_ (2.5%); THF–C_60_ (5%); THF–fish water (2.5%); THF–fish water (5%). Fish mortality was assessed 72 hr after exposure was initiated and behavior of fish in exposure solutions was recorded. Water quality characteristics (temperature, pH, and dissolved oxygen) were measured in treatments at initiation of the experiment, and values were within the range reported for fish water control (see above).

Two additional dose–response tests were conducted separately from experiments described above. The first test chemical was THF (d^20^_4_ = 0.8892) at the following concentrations (vol/vol): 0%, 0.04%, 0.08%, 0.16%, 0.31%, 0.63%, 1.25%, 2.5%, 5%, and 10%; and the second test chemical was γ-butyrolactone (d^15^_0_ = 1.1286) at the following test concentrations (vol/vol): 0%, 0.0001%, 0.0005%, 0.001%, 0.003%, 0.005%, 0.01%, 0.05%, and 0.1%. Procedures for these dose–response tests were identical to those described above.

### Total RNA extraction and microarray analyses

Pairs of the six replicate beakers for each treatment were combined to make three replicates for analysis of differential gene expression by microarray (three arrays per treatment). Total RNA was extracted from fish larvae that survived exposure using the RNA easy mini kit for animal tissues (Qiagen, Valencia, CA, USA). RNA was extracted from larvae using RLT buffer, purified using RNeasy columns, and included DNase digestion according to the procedure described in the RNeasy Mini Handbook for animal tissues (Qiagen 2006). Total RNA was processed by the Affymetrix Core Facility at the University of Tennessee (Knoxville, TN, USA) according to Affymetrix GeneChip Expression Analysis Technical Manual (Affymetrix 2004). Processing of total RNA included synthesis of cDNA (One-Cycle cDNA synthesis kit; Affymetrix, Santa Clara, CA, USA), biotin labeling (3′IVT Labeling Kit; Affymetrix), hybridization, and scanning according to standard Affymetrix protocols. For each zebrafish array, an equal amount of labeled cRNA (5 μg) was used. All arrays were assessed for quality measurements including assurance that IVT (*in vitro* transcription) glyceraldehyde-3-phosphate dehydrogenase (GADPH) 3′/5′ values were < 3, and all internal spike in controls were present at anticipated levels. The Affymetrix GeneChip Zebrafish Genome Array contains approximately 15,509 probe sets that represent 14,900 *D. rerio* gene transcripts. Probe sets for the array were designed by Affymetrix, members of the zebrafish community, and the National Institutes of Health investigators using public data sources: RefSeq (July 2003; http://www.ncbi.nlm.nih.gov/RefSeq/), GenBank (*Danio rerio* (zebrafish) genome; release 136.0, June 2003; http://www.ncbi.nlm.nih.gov/mapview/map_search.cgi?taxid=7955), dbEST (Expressed Sequence Tag; July 2003; http://www.ncbi.nlm.nih.gov/dbEST), and UniGene (Build 54, June 2003; http://www.ncbi.nlm.nih.gov/UniGene/UGOrg.cgi?TAXID=7955). Sixteen pairs of oligonucleotide 25-mer probes are used to measure the level of transcription of each sequence. Detection sensitivity is estimated at 1:100,000. Housekeeping and control genes are GADPH and alpha 1 actin. Hybridization controls included are *bioB*, *bioC*, *bioD*, and *cre*. The Affymetrix Probe Set ID and the gene title and symbol (UniGene; http://www.ncbi.nlm.nih.gov/sites/entrez?db=unigene) are reported for annotated genes along with their GenBank accession number (http://www.ncbi.nlm.nih.gov/Genbank/index.html).

### Data analysis

The 72-hr lethal concentration predicted to kill 50% of larval zebrafish (LC_50_) was computed by Trimmed Spearman-Karber method (version 1.5; U.S. Environmental Protection Agency 1993). Mortality of fish in exposures used for gene expression analyses was modeled by analysis of variance (ANOVA) after arcsin transformation of mortality data (Zar 1984), and significant (*p* < 0.05) differences among groups were assessed by Tukey test (Proc GLM, Statistical Analysis System, version 9.1; SAS Institute, Cary, NC).

Statistical assessment of differential gene expression data was conducted according to standard procedures by the University of Tennessee Affymetrix Core Facility. Intensity of expression of individual gene transcripts was obtained from scanned images of zebrafish arrays (Affymetrix 3000 7G) and signal values were obtained using the GCRMA algorithm (ArrayAssist; Stratagene La Jolla, CA, USA). Genes with a fold change in expression of < 1.75 were removed from the data set along with genes that had low signal intensity (< 16 on a scale of 65,536) indicative of minimal (i.e., background) expression. Expression of the resulting set of genes was assessed by an ANOVA model with Benjamini-Hochberg (false discovery rate, FDR) correction (ArrayAssist; Stratagene). After determination of significance (*p* < 0.05) of the overall ANOVA model, pairwise comparisons of each experimental treatment with the control were conducted by *t*-test.

## Results

### Water chemistry analyses

**Figure 2:**
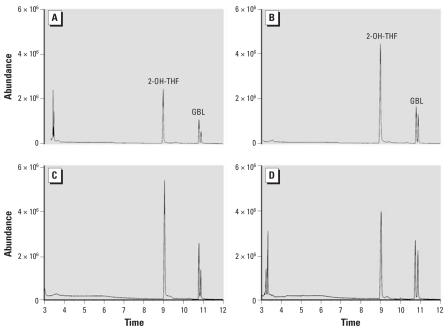
TIC traces obtained from GC-MS analysis of THF-treated water samples. Abbreviations: 2-OH-THF: tetrahydro-2-furanol; GBL, γ-butyrolactone. (*A*) THF, time = 0; (*B*) THF–C_60_, time = 0; (*C*) THF, time = 75 hr; (*D*) THF–C_60_, time = 75 hr.

**Figure 3:**
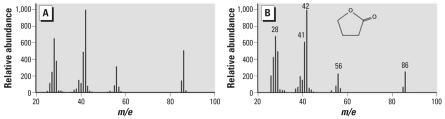
Mass spectrum of γ-butyrolactone obtained from (*A*) THF–C_60_-treated sample at 72 hr; (*B*) spectrum obtained from authentic standard. Intensity of each peak was adjusted to base peak (*m*/*e* 42).

Each water sample was analyzed for presence of THF and impurities, and THF was found in all samples collected immediately after vacuum extraction. However, in water that was sparged with nitrogen gas (N_2_) for 2.5 days after vacuum extraction, THF was not detected. γ-Butyrolactone and tetrahydro-2-furanol were detected in all THF-treated samples (Figure 2). Tetrahydro-2-furanol was confirmed by comparison with NIST Mass Spectral Library (2005) because authentic standard was not available, and γ-butyrolactone was determined by comparison with an authentic standard (Figure 3). Immediately after preparation, the concentration of γ-butyrolactone was approximately 103 ppm and approximately 158 ppm in THF–water and THF–C_60_ treatments, respectively, and after 72 hr, concentrations of γ-butyrolactone increased to approximately 164 ppm in THF–water and approximately 175 ppm in THF–C_60_. γ-Butyrolactone and tetrahydro-2-furanol were detected by (gas chromatography–mass spectrometry (GC-MS) in some freshly opened THF bottles prior to use in experiments.

### Larval fish survival

**Figure 4:**
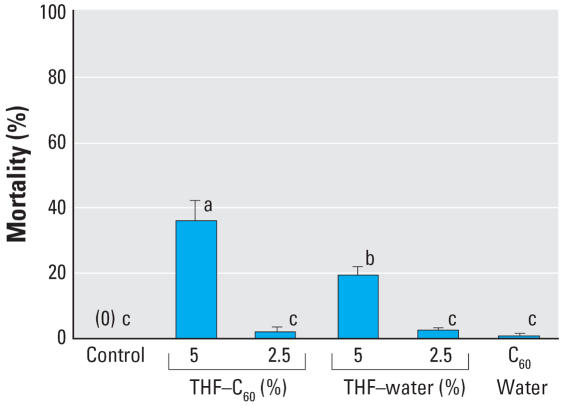
Mean mortality (± SD, *n* = 3) of larval zebrafish in control (fish water) and experimental treatments after 72-hr exposure. Larvae that survived exposure in the control, THF–C_60_ (2.5%), THF–water (2.5%) and C_60_–water treatments were used for microarray analyses. Significant (*p* < 0.05) differences in mortality among groups are indicated by different letters.

No fish died during 72-hr exposure in the control, and mortality increased with concentration in both THF–water and THF–C_60_ with LC_50_ values of 6.3% [95% confidence interval (CI), 5.1–7.8%] and 3.1% (95% CI, 2.3–4.2%) respectively. At higher concentrations (> 5%) in THF–water and THF–C_60_, lethal effects were observed within 60 min, and fish typically had arched backs and occasionally had severe yolk-sac and pericardial edema. In concentrations with significant partial mortality (i.e., 5% exposure concentration), surviving fish were lethargic, laterally recumbent, and typically had minor to severe yolk-sac and/or pericardial edema. Swimming movements were limited to abrupt spasms that occurred only when fish were disturbed. Fish exposed to concentrations of 1% appeared more lethargic than control fish but did not have any grossly visible indications of toxicity (e.g., tissue edema, or uncoordinated swimming). For larvae exposed in the experiment designed for analysis of gene expression, mortality was significantly higher in 5% THF–C_60_ than in 5% THF–water, and both treatments had significantly higher mortality than 2.5% THF–C_60_, 2.5% THF–water, and C_60_–water, which did not differ significantly from the control (Figure 4).

No control fish died in either THF or γ-butyrolactone 72-hr dose–response tests and mortality increased with concentration of each test chemical. For THF, the LC_50_ was 1.73% (95% CI, 1.65–1.81%), whereas the LC_50_ for γ-butyrolactone was 0.0047% (95% CI, 0.0038–0.0058%). Analyzed concentrations of test chemicals were within 10% of nominal concentrations in dose–response tests, and γ-butyrolactone was not detected in water samples in the dose–response test with THF.

### Gene expression

Gene expression was evaluated in total RNA samples from the control, C_60_–water, 2.5% THF–C_60_, and 2.5% THF treatments. The total numbers of genes in which significant changes in expression were detected relative to the control were 10 in C_60_–water, 217 in THF–water, and 271 in THF–C_60_. Array data files were uploaded to the Stanford microarray database installed at the University of Tennessee Microarray Database (UT-MD) and are available for public access (UT-MD 2007). Genes not assigned by gene ontology to a biological process, cellular component, or molecular function (unannotated genes) included 40–50% of the observed genes that were differentially expressed. Of the 10 genes differentially regulated in C_60_–water relative to control, only 2 (both unannotated) were up-regulated (Table 1).

**Figure 5:**
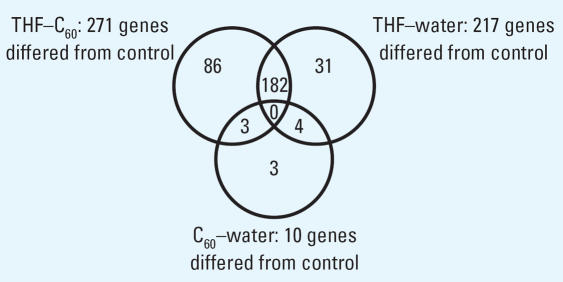
Numbers of genes in each treatment that differed significantly from the control. Numbers that fall into more than one circle were common to both treatments, whereas numbers in only one circle indicate genes that were unique for the specific treatment.

**Figure 6:**
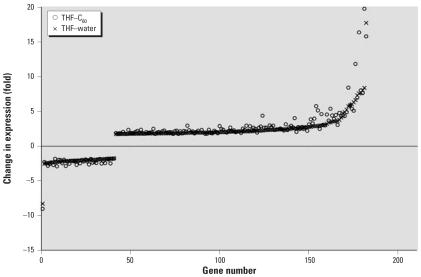
Fold changes in expression for the 182 common genes in larval zebrafish from THF–water and THF–C_60_ treatments with expression levels that differed significantly from the control. The *x*-axis is the number of the gene, which was ordered based on the fold change observed in THF–water.

Some of the same genes that were differently regulated relative to the control were found in multiple treatments (Figure 5). In C_60_–water, there were 4 genes in common with THF–water treatment and 3 genes in common with THF–C_60_ treatment; however, the magnitude and direction (i.e., up-regulation or down-regulation) of change in expression were not necessarily consistent (Table 1). For the 3 genes that were in common between C_60_–water and THF–C_60_, only one (Dr.3448.1.S1, Kruppel-like factor 2a, *klf2a*) had a similar change in expression relative to control (−1.85-fold, THF–C_60_; −1.78-fold, C_60_–water). The two THF treatments had 182 genes in common and change in expression of these genes was identical in direction but differed in magnitude (Figure 6). The magnitude of change in expression was higher for 133 of the 182 genes (73%) in THF–C_60_ than in THF–water. Of particular interest were highly up-regulated genes relative to control in both THF treatments that corresponded to enzymes with oxidoreductase and peroxidase activity (Table 2).

**Figure 7:**
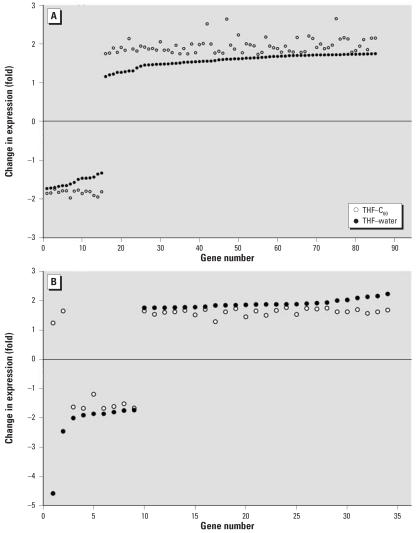
Change in expression level (fold change) for genes of larval zebrafish from THF–water and THF–C_60_ treatments relative to expression level of the control. The *x*-axis is the number of the gene, which was ordered based on the fold change observed for the THF–water. (*A*) The 89 genes from THF–C_60_ with expressions that were significantly different from the control, but which had expression levels between −1.75 and +1.75 in THF–water (thus not identified as significantly different from the control for THF). (*B*) The 35 genes from THF–water with expressions that were significantly different from the control, but which had expression levels between −1.75 and +1.75 in THF–C_60_ (thus not identified as significantly different from the control for THF–C_60_).

Both THF–water and THF–C_60_ treatments induced significant changes in gene expression in zebrafish; however, not all genes identified as significant were the same in each treatment relative to the control (Figure 5). In THF–C_60_, 89 genes differed significantly from control but did not differ in THF–water compared with control; however, examination of the expression of these 89 genes in THF–water indicated that direction of change in expression relative to the that in control was identical to THF–C_60_ (Figure 7A). The magnitude of change in expression for these 89 genes in THF–water relative to that in control was < 1.75-fold, and therefore these genes did not meet selection criteria established for statistical analyses. The same pattern was observed for 33 of the 35 genes that were recognized in THF–water as different from control, but that did not differ in THF–C_60_ compared with control (i.e., the magnitude of expression change was < 1.75-fold in THF–C_60_; Figure 7B). Only 2 genes exhibited a different pattern of expression change, and these 2 genes were down-regulated in THF–water but up-regulated in THF–C_60_ (genes 1 and 2; Figure 7B). When changes in gene expression were compared directly between THF–water and THF–C_60_, these 2 genes (Affymetrix probe set identification numbers: DrAffx.3.1.A1, DrAffx.1.85.A1) were the most up-regulated genes after exposure to THF–C_60_. Expression of a third unannotated gene (Dr.955.1.A1, *Wu:fb98b06*) differed between THF–C_60_ and THF–water but differences in expression were not detected for this gene when either treatment was compared with control. Four genes (Dr.10914.1.A1; Dr.15281.1.A1, tissue inhibitor of metalloproteinase 2–like, *timp2l*; Dr.967.1.S1, matrix metalloproteinase 9, *mmp9*; Dr.10314.1.S1, matrix metalloproteinase 13, *mmp13*) were up-regulated in both treatments relative to control, but up-regulation was higher in THF–C_60_ and expression of these genes differed significantly when compared with THF–water.

## Discussion

Survival of zebrafish larvae was affected only in THF–C_60_ and THF–water and was not affected in C_60_–water or in the control. Mortality of fish in THF treatments was concentration dependent. Analyses of water samples by GC-MS did not detect THF, and the 72-hr acute toxicity of THF determined in the dose–response test indicated that THF is of low toxicity in larval zebrafish (LC_50_ = 1.73%; ~ 15.4 g/L). Thus, any trace amounts of THF in THF–C_60_ and THF–water treatments did not explain the observed fish mortality. However, use of THF as a vehicle resulted in generation of substances in the water that were detected by GC-MS and could be responsible for toxic effects. Notable among these substances was γ-butyrolactone, which was shown to be acutely toxic at low concentrations (72-hr LC_50_, 0.0047%; ~ 47 mg/L based on dilution of analyzed prepared solution) in larval zebrafish in this study. Estimated concentrations of γ-butyrolactone (based on dilution of the analyzed prepared solution) at the LC_50_ for THF–water and THF–C_60_ treatments were 10 and 5 mg/L, respectively, at the end of exposure.

γ-Butyrolactone is a colorless liquid that is soluble in water (Ciolino et al. 2001) and rapidly absorbed in mammals where it is readily converted into the neurotransmitter gamma amino butyric acid (GABA) in several steps by enzyme action (Bernasconi et al. 1999). In zebrafish, GABA receptors are responsive within 96 hr of fertilization (Saint-Amant and Drapeau 2000) and may be involved in control of ventilatory movements during early larval zebrafish development (Turesson et al. 2006). The toxicity of γ-butyrolactone in zebrafish has not been investigated previously; however, unpublished data indicate acute (48 hr) toxicity in fish (golden orfe *Leuciscus idus*) with LC_50_ of 275–302 mg/L and in the daphnid *Daphnia magna* with an LC_50_ of > 500 mg/L (Lyondell Chemical Company 2004).

The present research is the first to demonstrate that compounds other than THF or C_60_ may be responsible for toxicity observed in treatments in which THF was used as a vehicle for C_60_. Toxic effects of THF–C_60_ have been observed in largemouth bass *Micropterus salmoides* (Oberdörster 2004), *D. magna* (Lovern and Klaper 2006), and in cultured cell lines (Sayes et al. 2004). The procedure we used to prepare THF–C_60_ described here differs from previously published methods in the manner that THF was removed from the sample and assessment of any residual THF. Previous studies (e.g., Lovern and Klaper 2006; Oberdörster 2004) used two sequential rotovaping and water suspension cycles followed by filtration prior to preparation of C_60_ dilutions. Complete removal of THF was assumed and analysis for THF or THF degradation products was not performed. In the present method, subsequent to rotovaping, samples were sparged with N_2_ for 2.5 days to ensure THF removal. Samples were analyzed with GC-MS and although no residual THF was found, THF degradation products were found. Despite the effectiveness of THF removal, THF degradation products were retained in the sample. Although it is conjectural to make statements about the previously published method because GC-MS was not performed, it is unlikely that all THF products were removed using that method. In the present study we demonstrate that THF degradation products can contribute to toxicity in THF–C_60_ solutions and previous research (Lovern and Klaper 2006; Oberdörster 2004; Sayes et al. 2004) cannot exclude that THF or THF degradation products did not contribute to toxic effects. It is interesting to note that the particle diameter of C_60_ aggregates showed no significant difference between filtered and nonfiltered samples under the same sample preparation technique (Cheng et al. 2006).

Most relevant to the present investigation is research in largemouth bass (Oberdörster 2004), which indicated oxidative injury in the brain resulted after exposure to THF–C_60_. Oberdörster (2004) directly determined lipid peroxidation in the brain by the thiobarbituric acid (TBA) assay for malondialdehyde and found higher peroxidation of lipids in fish exposed to THF–C_60_. We did not investigate oxidative injury directly in zebrafish, but analyses of gene expression can provide important insight on organism physiology and be used to deduce affected metabolic pathways (Brazma et al. 2001). Up-regulation of genes with antioxidant activity (including glutathione *S*-transferase) in THF–water and THF–C_60_ treatments in the present study is consistent with the hypothesis that fish were responding to defend against oxidative injury resulting from exposure to oxidative chemicals. Oxidative injury in the brains of large-mouth bass observed by Oberdörster (2004) could have resulted from oxidative substances generated by THF vehicle rather than C_60_ directly.

Changes in global gene expression were nearly identical in THF–water and THF–C_60_ treatments, and these results indicate that fish were responding to similar exposure scenarios in both treatments. Although expression of specific genes that had significant changes in expression could be probed further with additional techniques [e.g., quantitative reverse transcriptase polymerase chain reaction (qRT-PCR)] to clarify how a treatment affects particular genes, changes in expression appear related to the presence of THF or THF degradation products rather than C_60_. Therefore, further pursuit of these genes (e.g., genes listed in Table 2) as bio-markers of exposure to C_60_ was not warranted.

THF–C_60_ was more toxic than THF–water based on larval zebrafish survival (5% concentration) and gene expression patterns (2.5% concentration). The magnitude of the change in gene expression was higher in THF–C_60_ for 73% of the 182 genes that were in common between THF treatments, and of the 124 genes that differed separately from the control (89 genes THF–C_60_, 35 genes THF–water) 72% had a higher magnitude of expression change in THF–C_60_. These results may be explained by either higher concentrations of γ-butyrolactone (or other THF degradation products) in THF–C_60_, by presence of C_60_, or possibly by an interaction between the C_60_ and γ-butyrolactone (or other THF degradation products). The concentration of γ-butyrolactone was higher in THF–C_60_ (stock solutions after preparation: ~ 158 ppm in THF–C_60_; ~ 103 ppm in THF–water); this could have resulted from differences in evaporation of water during preparation (particularly during N_2_ sparging) or, perhaps, the presence of C_60_ enhanced the formation of γ-butyrolactone. This investigation was not designed to determine if C_60_ could influence formation of γ-butyrolactone; however, further investigation into this possibility is warranted.

Larval zebrafish exposed to C_60_ (C_60_–water treatment) did not die as a result of exposure, and gene expression changes relative to the control were relatively minimal. Of the 10 genes with altered expression, only 2 genes were up-regulated and the function of these genes was unknown (unannotated). Further investigation is required to determine if these two up-regulated genes indicate exposure to aqueous C_60_ aggregates and the physiologic importance of those biochemical pathways. For the 8 down-regulated genes, 3 were related by electronic annotation to a known function. Modulation of the heat-shock gene indicates a general stress response; however, down-regulation of this gene is difficult to interpret. Similarly, down-regulation of the gene that codes for an RNA binding factor and the gene with homology to a Kruppel-like factor (KLF) is difficult to interpret but could suggest a global modulation of transcription in response to C_60_ exposure (although changes in global modulation of transcription is not supported by changes in expression in other genes). The KLF family of transcription factors bind to Sp1 sequences, modulations of which have been implicated in growth-related signal transduction pathways as well as apoptosis, angiogenesis, and tumorgenesis (Black et al. 2001). The rather minimal change in global gene expression observed when zebrafish larvae were exposed to C_60_–water indicates that the exposure scenario used in this investigation had only minimal effects on the fish. Longer exposures to C_60_ or other exposure scenarios (different species, life history stages) may result in different effects. A final point is as that changes in gene expression were investigated after a 72-hr exposure, presumably the expression of these genes (and likely other genes) will be affected differently after different exposure durations and at different zebrafish life history stages.

## Figures and Tables

**Table 1:** Differentially expressed genes identified by microarray analyses of larval zebrafish after exposure to C_60_–water in comparison to control.

Affymetrix Probe set ID	Gene title (*gene symbol*)	GenBank accession no.^a^	Fold difference	*p*-Value	Description/function
DrAffx.3.1.A1^b^	CDNA clone MGC:113968	BX296557.35	−6.29	0.019	Transport, nuclear pore
DrAffx.1.85.A1^c^		AY325265.1	−2.44	0.005	Unknown
Dr.2619.1.S1^d^	RNA binding motif protein, X-linked (*rbmx*)	AL929434.3	−2.22	0.006	Nucleotide binding
Dr.6431.1.S1^e^	zgc:56537 (*zgc:56537*)	NM_199950.1	−2.10	0.001	Intracellular signaling
Dr.691.1.S1^f^	zgc:101565 (*zgc:101565*)	NM_001004641.1	−1.90	0.005	Metabolism, oxidoreductase
Dr.25536.1.A1^g^	Similar to Heat shock protein HSP 90-alpha (HSP 86) (*DKEY-241L7.8*)	CR381646.8	−1.86	0.000	Unknown
Dr.3448.1.S1^h^	Krupple-like factor 2a (*klf2a*)	NM_131856.1	−1.78	0.045	Nucleic acid binding
Dr.20524.1.A1	wu:fj52d04 (*wu:fj52d04*)	NM_001045321.1	−1.75	0.029	Unknown
Dr.26327.1.A1	DiGeorge syndrome critical region gene 8 (*dgcr8*)	BX088551.7	1.90	0.023	Unknown
Dr.7842.1.A1	wu:fj15c09 (*wu:fj15c09*)	CR548643.7	2.10	0.001	Unknown

a Accession numbers are from GenBank (http://www.ncbi.nlm.nih.gov/Genbank/). Genes were also differentially regulated relative to control for THF–water treatment; fold changes were

b −4.53,

c −2.29,

d −1.88,

f −1.76. Genes also differentially regulated relative to control for THF–C_60_ treatment; fold changes were

e 1.76,

g −1.86,

h −1.78.

**Table 2:** Selected up-regulated genes in larval zebrafish in THF–water and THF–C_60_ compared to control. No significant changes in expression were observed for these genes between THF–C_60_ and THF–water treatments.

		Expression change (fold)		
Affymetrix Probe Set ID	Gene title (*gene symbol*)	THF–C_60_	THF–water	Description/function
Dr.10624.1.S1^a^	zgc:110343 (*zgc:110343*)	7.00	7.32	Peroxidase activity, antioxidant activity
Dr.23788.1.S1^b^	Glutathione S-transferase (*gstp1*)	5.39	6.05	Metabolism, glutathione transferase activity
Dr.9492.1.A1^c^	zgc:113156, hypothetical protein (*zgc:113156*)	3.69	3.43	Oxidoreductase activity

GenBank accession numbers (http://www.ncbi.nlm.nih.gov/Genbank/):

a AL954171.10;

b NM_131734.3, AF285098.1;

c XM_696730.1, NM_001017881.1.
